# Comparing olive oil and C4-dietary oil, a prodrug for the GPR119 agonist, 2-oleoyl glycerol, less energy intake of the latter is needed to stimulate incretin hormone secretion in overweight subjects with type 2 diabetes

**DOI:** 10.1038/s41387-017-0011-z

**Published:** 2018-01-12

**Authors:** Mette Johannsen Mandøe, Katrine Bagge Hansen, Johanne Agerlin Windeløv, Filip Krag Knop, Jens Frederik Rehfeld, Mette Marie Rosenkilde, Jens Juul Holst, Harald Severin Hansen

**Affiliations:** 1 The Novo Nordisk Foundation Center for Basal Metabolic Research, Faculty of Health and Medical Sciences, University of Copenhagen, Copenhagen, Denmark; 2 Department of Biomedical Sciences, Faculty of Health and Medical Sciences, University of Copenhagen, Copenhagen, Denmark; 3 Department of Clinical Physiology and Nuclear Medicine, Glostrup Hospital, University of Copenhagen, Glostrup, Denmark; 4 Center for Diabetes Research, Gentofte Hospital, University of Copenhagen, Hellerup, Denmark; 5 Department of Clinical Medicine, Faculty of Health and Medical Sciences, University of Copenhagen, Copenhagen, Denmark; 6 Department of Clinical Biochemistry, Rigshospitalet, University of Copenhagen, Copenhagen, Denmark; 7 Department of Neuroscience and Pharmacology, Faculty of Health and Medical Sciences, University of Copenhagen, Copenhagen, Denmark; 8 Department of Drug Design and Pharmacology, Faculty of Health and Medical Sciences, University of Copenhagen, Copenhagen, Denmark

## Abstract

**Background/objective:**

After digestion, dietary triacylglycerol stimulates incretin release in humans, mainly through generation of 2-monoacylglycerol, an agonist for the intestinal G protein-coupled receptor 119 (GPR119). Enhanced incretin release may have beneficial metabolic effects. However, dietary fat may promote weight gain and should therefore be restricted in obesity. We designed C4-dietary oil (1,3-di-butyryl-2-oleoyl glycerol) as a 2-oleoyl glycerol (2-OG)-generating fat type, which would stimulate incretin release to the same extent while providing less calories than equimolar amounts of common triglycerides, e.g., olive oil.

**Subjects and methods:**

We studied the effect over 180 min of (a) 19 g olive oil plus 200 g carrot, (b) 10.7 g C4 dietary oil plus 200 g carrot and (c) 200 g carrot, respectively, on plasma responses of gut and pancreatic hormones in 13 overweight patients with type 2 diabetes (T2D). Theoretically, both oil meals result in formation of 7.7 g 2-OG during digestion.

**Results:**

Both olive oil and C4-dietary oil resulted in greater postprandial (*P* ≤ 0.01) glucagon-like peptide 1 (GLP-1) and glucose-dependent insulinotropic polypeptide (GIP) responses (incremental area under curve (iAUC)): iAUC_GLP−1_: 645 ± 194 and 702 ± 97 pM × min; iAUC_GIP_: 4,338 ± 764 and 2,894 ± 601 pM × min) compared to the carrot meal (iAUC_GLP−1_: 7 ± 103 pM × min; iAUC_GIP_: 266 ± 234 pM × min). iAUC for GLP-1 and GIP were similar for C4-dietary oil and olive oil, although olive oil resulted in a higher peak value for GIP than C4-dietary oil.

**Conclusion:**

C4-dietary oil enhanced secretion of GLP-1 and GIP to almost the same extent as olive oil, in spite of liberation of both 2-OG and oleic acid, which also may stimulate incretin secretion, from olive oil. Thus, C4-dietary oil is more effective as incretin releaser than olive oil per unit of energy and may be useful for dietary intervention.

## Introduction

Weight loss is an important goal in the treatment of obesity and diabetes, and can be achieved by a lower intake of dietary fat as it is known that a higher dietary fat intake is associated with weight gain.^1^ Furthermore, the drug orlistat,^2^ which decreases fat absorption, as well as substitution of long-chain triglycerides with medium-chain triglycerides in the diet^3^ are known to produce weight loss in humans. In a human weight loss regime, dietary fat restriction was, calorie for calorie, more efficient than carbohydrate restriction with respect to body fat loss,^4^ supporting substitution of some of the fat in the human diet with carbohydrate or protein for prevention of weight gain. However, dietary fat is a potent stimulus for incretin release,^5^ which appears to have several beneficial functions in energy metabolism.^6^ Upon digestion of dietary fat, it is hydrolyzed to 2-monoacylglycerol and fatty acids, which both are absorbed in the small intestine, re-esterified and exported to the circulation with chylomicrons. It turns out that it is the hydrolysis products that stimulate incretin release,^7–10^ although recent results suggest that fatty acids may stimulate glucagon-like peptide 1 (GLP-1) release from the vascular site and not the luminal site.^11^ At a dose of dietary fat reflecting a light meal, e.g., 20 ml olive oil (21.6 mmol), it appears that it is mainly the 2-monoacylglycerol moiety of the fat that is responsible for stimulating incretin release, whereas the fatty acid moieties may have a minor role.^12^ This conclusion was reached from studies of a C8-dietary oil, 1,3-di-octanoyl-2-oleoyl glycerol, which upon digestion results in formation of two molecules of a medium-chain fatty acid, octanoic acid, and 2-oleoyl glycerol (2-OG). Octanoic acid is a poor agonist for fatty acid receptors,^13^ and dietary medium-chain triglycerides are poor stimulators of gut hormone release,^12, 14^ while 2-OG alone readily stimulates release of incretins via activation of the G protein-coupled receptor 119 (GPR119).^12, 15, 16^ Thus, on a molar basis the C8-dietary oil has the same potency as long-chain-triglycerides, although it provides only 61% of the calories, i.e., for 21.6 mmol, 107 kcal versus 171 kcal.^12^ However, prolonged intake of octanoic acid may have the disadvantage of stimulating the formation of bioactive *N*-octanoyl-ghrelin,^17, 18^ which may promote food intake. Thus, we have chosen to study another oil in which octanoic acid is substituted by butyric acid in a similarly structured triacylglycerol, i.e., 1,3-dibutyryl-2-oleoyl-glycerol, henceforth designated C4-dietary oil, which also contains even less calories (81 kcal for 21.6 mmol) on a molar basis. From mouse studies, butyric acid is known to be able to elevate proglucagon gene expression in the intestine,^19^ increase the number of enteroendocrine L cells,^20^ improve vascular function and inhibit inflammation, probably via inhibition of histone deacetylases involved in epigenetic regulations.^21, 22^ Furthermore, tributyrin/butyrate had anti-obesity and anti-diabetic effects after prolonged feeding in mice^23–25^ as also seen with microbiota-derived short-chain fatty acids; the latter apparently exerted their anti-obesity effect via GPR43 activation.^24^ Targeted delivery of another short-chain fatty acid, propionate, had beneficial effect on body weight maintenance and adiposity in overweight adults.^26^ Butyric acid was reported to stimulate GLP-1 release from murine L cells,^27^ although an intake of 6.53 g tributyrin did not stimulate incretin release in humans.^12^

In the present study, we tested whether intake of 10.7 g C4-dietary oil (expected to generate 7.7 g 2-OG) stimulates incretin release in 13 overweight diabetic subjects. The results were compared to those obtained after intake of 19 g (20 ml) olive oil, which provides both long-chain fatty acids and 2-OG (7.7 g equal to 21.6 mmol) and many more calories (177 versus 81 kcal) or compared to a carrot meal alone.

## Material and methods

The pro-drug, C4-dietary oil, was first tested as an agonist as compared to the well-described agonist oleoylethanolamide (OEA)^28^ for transiently expressed human GPR119 in COS-7 cells, and subsequently in 13 patients with type 2 diabetes (T2D) in a randomized, single-blinded study. We compared the effect of C4-dietary oil (active compound), olive oil (active comparator) on a background of grated carrot (“placebo”) administered orally as three different “meals” in a cross-over design. The two oils were consumed in a shot-glass while eating the grated carrot. The primary outcome was postprandial plasma responses of GLP-1 measured during and 3 h after ingestion of the different meals (assessed as incremental area under the curve (iAUC)). Secondly, we investigated the effects of the meals on glucose-dependent insulinotropic polypeptide (GIP), on insulin and glucagon release from the pancreas, and on the appetite-regulating gut hormones peptide YY (PYY) and cholecystokinin (CCK).

### In vitro studies

#### GPR119 signaling

COS-7 cells were grown at 10% CO_2_ and 37 °C (310 K) in Dulbecco’s modified Eagle’s medium supplemented with GlutaMAX™ (Life Technologies Corporation, Carlsbad, CA, USA), 10% fetal bovine serum, 180 IU/ml penicillin and 45 µg/ml streptomycin (PenStrep) and were transfected by the calcium phosphate precipitation method.^29^ The cAMP formation was determined by the HitHunter cAMP XS+assay (DiscoveRx, Fremont, CA, USA).^30^ In brief, the transiently transfected cells were seeded into 96-well plates (35,000 cells/well). Twenty-four hours later, cells were washed twice with HBS buffer (20 mM HEPES, 150 mM NaCl, pH 7.4) and incubated with ligands (C4-dietary oil (Larodan, Malmø, Sweden) or OEA (Cayman Chemicals, Ann Harbor, MI, USA)) for 30 min at 37 °C in HBS containing 1 mM isobutylmethylxanthine phosphodiesterase inhibitor (Sigma-Aldrich, St. Louis, MO, USA). For the in vitro experiment C4-dietary oil was dissolved in 50% DMSO and then diluted with medium to the desired concentrations, having a final concentration of DMSO of 1%. After incubation, the medium was removed and the cells were treated according to the protocol for the “three reagent addition” procedure using the HitHunter cAMP XS+assay, an enzyme fragment complementation-based cAMP assay. The amount of cAMP was measured as luminescence using Perkin Elmer EnVision 2104 Multilabel Reader (Walton, MA, US). Determinations were made in triplicate.

### In vivo studies

#### Ethical approval

The study was conducted according to the principles of the Helsinki Declaration II and approved by the Scientific-Ethical Committee of the Capital Region of Denmark (Registration Number H-3-2011-007).

#### Subjects

Thirteen overweight Caucasians patients (eight males, five females) diagnosed with T2D (according to World Health Organization criteria^31^) participated. All patients were recruited from diabetes outpatient clinics in order to reflect clinical reality. Patient characteristics are listed in Table 1. All participants had negative tests of islet cell auto-antibodies and glutamate decarboxylase 65 auto-antibodies. Exclusion criteria included kidney disease with serum creatinine >130 µM and/or albuminuria, liver disease with plasma alanine transaminase >twice the upper limit of the normal range, diabetic neuropathy, proliferative retinopathy, anemia, treatment with insulin, GLP-1 receptor agonist and/or dipeptidyl peptidase 4 (DPP-4) inhibitor and inability to pause medication for at least 10 h. The subjects had a ‘low’ HbA1c but they paused their antidiabetic medication (biguanide or sulfonylurea) 7 days prior to each test day and therefore present with a fairly high fasting blood glucose level. All subjects agreed to participate by signing an informed consent after receiving oral and written information prior to procedures. The participants were identified by a study protocol identification number.

**Table 1:** Patient characteristics

Male/female	8/5
Age (years)	65 (47–75)
Body weight (kg)	91.9 ± 16.5
BMI (kg/m^2^)	30.0 ± 4.3
Fasting PG (mM)	9.0 ± 3.6
HbA1_c_ (%)	6.9 ± 1.3
Fam. history of diabetes	8
Years of diabetes	4 (0.5–11)

Data are numbers (gender) or mean values ± standard deviation SD or with range in parentheses (age and years of diabetes)

*BMI* body mass index, *FPG* fasting plasma glucose, *HbA1c* glycated hemoglobin A1c

#### Study design

The study was designed as a randomized, single-blinded cross-over study. It was blinded for the patients and analyst but it was not possible to blind it for the staff handling the different oils. The subjects were studied in a recumbent position after an overnight (10 h) fast including water, medication and tobacco abstinence. A cannula was inserted into a cubital vein. The cannulated arm was wrapped in a heating pad throughout the experiment for collection of arterialized blood samples. On three different days and in random order the participants received the different test meals, consisting of 200 g grated carrot and 1.0 g acetaminophen dissolved in 40 ml water plus a shot glass of either (a) 19 g of olive oil, or (b) 10.7 g of diet oil, or (c) no addition. Acetaminophen was added in order to measure gastric emptying rate. Two-hundred grams carrot was used as vehicle since it had no measurable effect on incretin hormone concentrations in peripheral plasma.^12^ There were 2–10 days between the different study days in order to avoid too much baseline variation. Theoretically, both oil “meals” would result in formation of 7.7 g 2-OG during digestion. The caloric content of the oils was 171 kcal (olive oil) and 81 kcal (C4-dietary oil), respectively, calculated on the basis of the amount of ATP generated from total oxidation of the two oils, and the notion that dietary fat contains 9 kcal/g. As shown in the figures arterialized blood was drawn at specific timepoints after ingestion of the meal and dispensed into chilled tubes containing EDTA plus aprotinin (500 KIU/ml blood; Trasylol^®^, Bayer Corp., Leverkusen, Germany) and a specific DPP-4 inhibitor (valine-pyrrolidide (0.01 mM); a gift from Novo Nordisk, Bagsværd, Denmark) for analysis of glucagon, GIP, GLP-1, PYY, CCK and triglycerides. For insulin and C-peptide analyses, blood was sampled into chilled tubes containing heparin. All tubes were kept on ice before and after blood sampling. They were centrifuged for 20 min at 1.500*g* and 4 °C. Plasma for glucagon, GLP-1, GIP, PYY, CCK and triglyceride analyses was stored at −20 °C and plasma for insulin and C-peptide analyses was stored at −80 °C until analysis. For analysis of acetaminophen, blood was distributed into dry vials and left to coagulate for 20 min at room temperature before centrifugation and handling as described below. For bedside measurement of plasma glucose (PG), blood was collected in fluoride tubes and centrifuged (7.400*g*) immediately for 2 min at room temperature.

#### Solutions

Olive oil (Frantoio 100% italiano, non filtrato, olio extra vergine di oliva) was from the manufacturer “Rocchi” (Oleificio R.MSant’Alessio Lucca, Italy). C4-dietary oil (1,3-dibutyryl 2-oleoyl glycerol of > 97% purity, with impurities being mainly 1,2-dibutyryl 3-oleoyl glycerol) was from Larodan (Malmø, Sweden). 1,3-dibutyryl 2-oleoyl glycerol is a natural constituent of milk fat.^32^

#### Analyses

Plasma samples were assayed for total GLP-1 immunoreactivity, as previously described,^33^ using antiserum no. 89390, which is specific for the C-terminal of the GLP-1 molecule. Intact, biologically active GIP was measured using antiserum no. 98171 as previously described.^34^ PYY(3–36) was measured using a commercially available radioimmunoassay kit from Millipore (catalog no. PYY-67HK; Billerica, MA, USA). The detection limit was 5 pM. The assay shows no cross-reaction with PYY(1–36) up to 250 pM. All quality controls were within prespecified limits. The concentrations of PG were measured bedside by the glucose oxidase method using a glucose analyzer (YSI model 2300 STAT plus analyzer; Yellow Springs Instrument Inc., Yellow Springs, OH, USA). Plasma C-peptide and insulin concentrations were measured using two-sided, electrochemiluminescense immunoassays (Roche/Hitachi modular analytics; Roche Diagnostic GmbH, Mannheim, Germany). The detection limit was less than 2 pM for both assays, and intra-assay coefficients of variation were 4.6% for the C-peptide assay and 1.9% for the insulin assay.^35^ The glucagon assay is directed against the C terminal of the glucagon molecule (antibody code no. 4305) and therefore measures glucagon of mainly pancreatic origin.^36–38^ CCK was measured as described earlier using antiserum no. 92128, which binds all bioactive forms of CCK and displays no cross-reactivity with gastrin.^39^ Triglycerides were measured using an enzymatic kit from Sigma-Aldrich (catalog no. TR0100; Saint Louis, MO, USA) with a modified protocol using 2 µl of plasma. Enzymatic reactions were performed in duplicates and measured in 96-well plates at 550 nm. Triglyceride concentrations were calculated according to the protocol. The working range was from 0.7 mM to 11.3 mM defined by a recovery of at least 80% and a coefficient of variation of <10%. Serum samples were assayed for acetaminophen using Vitros Chemistry Systems (Otho Clinical Diagnostics, Johnson & Johnson, Buckinghamshire, UK).

#### Statistical analyses and calculations

All results are expressed as means ± SEM unless otherwise mentioned. Statistical analysis was performed using Prism Graphpad 4 software (GraphPad Software, Inc., CA, USA). Repeated measurements ANOVA was followed by Tukey post hoc test. Area under curve (AUC) values were calculated using the trapezoidal rule and are presented as the incremental (area below baseline subtracted) AUC (iAUC) values if nothing else is stated in order to adjust for different baseline values. Furthermore, we analyzed differences in individual time points using paired *t* tests. The acetaminophen absorption test was used to determine gastric emptying rates^40^ calculated from tAUC.

## Results

### In vitro data

**Fig. 1:**
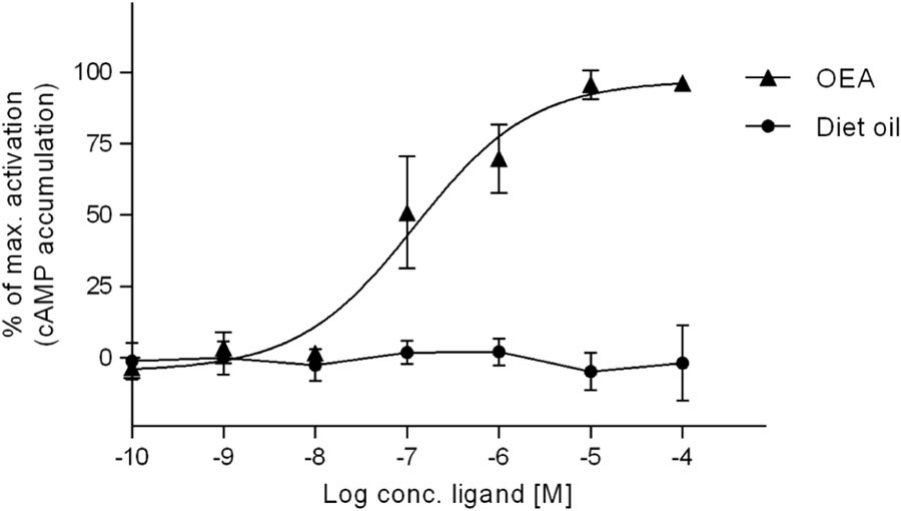
In vitro. C4-Dietary-oil does not by itself activates GPR119 transiently expressed in COS-7-cells Formation of cAMP was measured after stimulating GPR119-expressing COS-7 cells with oleoylethanolamide (OEA) and C4-dietary oil. The response is shown as percentage between full stimulation with OEA (100%) and no stimulation (0%) (*n* = 3)

The study on the COS-7 cells shows as expected no effect of C4-dietary oil in vitro (Fig. 1), in contrast to the potent stimulation by OEA with a half maximal effective concentration (EC_50_) of 1.2 × 10^−7^ M.

### In vivo data

Fourteen participants were initially recruited of whom one did not complete the study. This participant was excluded the 1st day of the study, because he was unable to eat 200 g carrot and vomited during ingestion of C4-dietary oil.

#### GLP-1, GIP and PYY

**Fig. 2:**
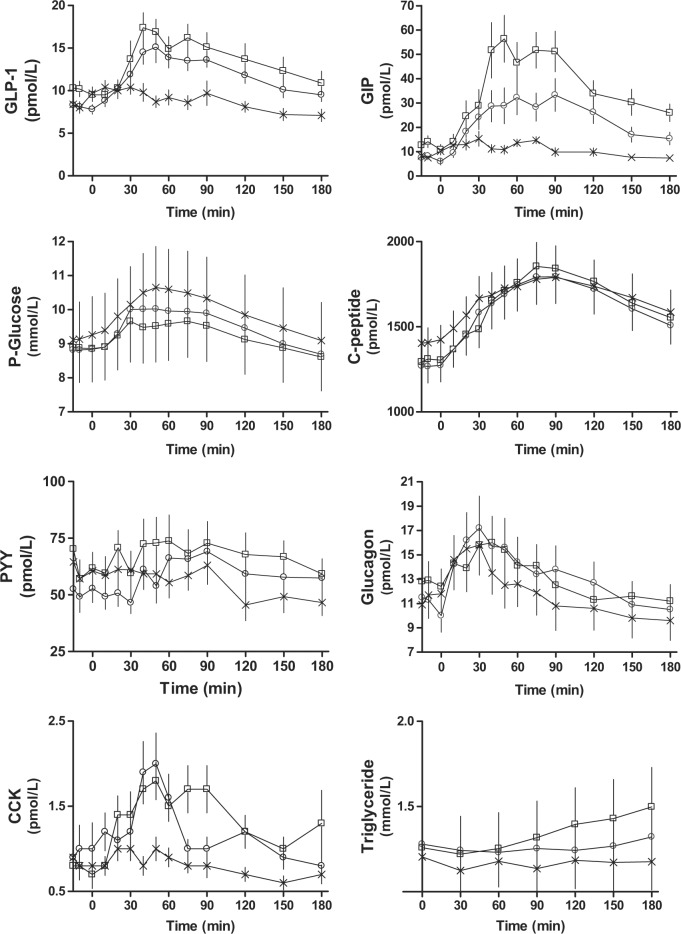
Time courses for plasma GLP-1, GIP, plasma glucose, glucagon, c-peptide, triglycerides, PYY and CCK following ingestion of olive oil and carrot (squares), C4-dietary oil and carrot (circles) and carrot alone (crosses) The three time points prior to ingestion was used in order to determine baseline and are only included as one point-value

Baseline plasma values for GLP-1 (olive oil: 10.0 ± 0.9 pM, C4-dietary oil: 8.1 ± 0.5 pM and carrot: 8.7 ± 0.6 pM. *P* = 0.01) and GIP (olive oil: 12.5 ± 2.1 pM, C4-dietary oil: 7.3 ± 1.2 pM, carrot: 8.6 ± 1.0 pM. *P* = 0.02), respectively, differed slightly on the different study days, while there was no difference for PYY (Table 2). Ingestion of both olive oil and C4-dietary oil in combination with carrot elicited greater GLP-1 (olive oil: 645 ± 194 pM × min, C4-dietary oil: 702 ± 97 pM × min, carrot: 7 ± 103 pM × min. *P* = 0.002) and GIP (olive oil: 4338 ± 764 pM × min, C4-dietary oil: 2894 ± 601 pM × min, carrot: 266 ± 234 pM × min. *P* < 0.0001) responses compared to the meal consisting of only carrot, and there were no differences in iAUC between olive oil-day and C4-dietary oil-day (Table 3). The peak value for GLP-1 was not different between the oil-days, whereas the peak value for GIP was higher for the olive oil-day than for the C4-dietary oil-day (olive oil: 68.85 ± 9.24 pM, C4-dietary oil: 38.92 ± 7.10 pM, carrot: 19.54 ± 2.46 pM. *P* < 0.0001 (Table 4)). iAUC and peak values for PYY were similar on the 3 days (Fig. 2, Table 3).

**Table 2:** Baseline values

	Olive oil (a)	C4-Dietary oil (b)	Carrot (c)	*P* value
GLP-1	10.0 ± 0.9	8.1 ± 0.5	8.7 ± 0.6	0.01^a>b^
GIP	12.5 ± 2.1	7.3 ± 1.2	8.6 ± 1.0	0.02^a>b^
PYY	63.1 ± 7.1	52.7 ± 5.5	58.1 ± 7.9	0.51
Glucose	8.9 ± 0.9	8.8 ± 1.0	9.2 ± 1.1	0.28
C-peptide	1,302 ± 79	1,270 ± 99	1,412 ± 86	0.11
Insulin	123 ± 15	113 ± 12	146 ± 16	0.05
Glucagon	12.7 ± 1.5	12.1 ± 1.3	12.6 ± 1.5	0.80
CCK	0.8 ± 0.2	1.0 ± 0.3	0.8 ± 0.2	0.34
Triglycerides	1.3 ± 0.2	1.3 ± 0.2	1.2 ± 0.2	0.86

Data are mean values ± SEM. All values in pM except glucose in mM. *P* values were calculated using repeated measurement ANOVA. *P* values ≤ 0.05 are considered statistically significant. Statistical significant differences between the three treatments are shown using the letters a, b and c, referring to the treatments

*CCK* cholecystokinin, *GIP* glucose-dependent insulinotropic polypeptide (intact), *GLP-1* glucagon-like peptide-1 (total), *PYY* peptide YY

**Table 3:** Area under curve (AUC)

	Olive oil (a)	C4-Dietary oil (b)	Carrot (c)	*P* value
GLP-1 iAUC	645 ± 194	702 ± 97	7 ± 103	0.002^a>c, b>c^
GLP-1 tAUC	2,580 ± 204	2,247 ± 115	1,698 ± 115	0.0002^a>c, b>c^
GIP iAUC	4,338 ± 764	2,894 ± 601	266 ± 234	<0.0001^a>c, b>c^
GIP tAUC	6,891 ± 1068	4,300 ± 765	2,055 ± 243	<0.0001^a>b, a>c, b>c^
PYY iAUC	855 ± 1,009	1,402 ± 581	−612 ± 628	0.21
PYY tAUC	13,110 ± 1,526	11,430 ± 1,344	10,650 ± 1,269	0.02^a>c^
Glucose iAUC	61 ± 26	172 ± 77	148. ± 38	0.23
Glucose tAUC	1,791 ± 197	1,837 ± 213	1,927 ± 225	0.04^c>a^
C-peptide iAUC	66,050 ± 10,800	66,310 ± 9,302	49,130 ± 10,800	0.08
C-peptide tAUC	320,100 ± 20,830	314,000 ± 25,310	324,400 ± 24,840	0.78
Insulin iAUC	8,438 ± 2,222	10,690 ± 1,671	4,901 ± 1,883	0.02^b>c^
Insulin tAUC	32,430 ± 2,220	32,580 ± 3,451	33,430 ± 3,378	0.92
Glucagon iAUC	17 ± 104	420 ± 95	18 ± 83	0.005^b>a, b>c^
Glucagon tAUC	2,513 ± 278	2,556 ± 350	2,292 ± 343	0.11
CCK iAUC	103 ± 34	33 ± 37	−11 ± 22	< 0.01^a>c^
CCK tAUC	252 ± 29	229 ± 27	151 ± 19	0.001^a>c, b>c^
Triglycerides iAUC	12.0 ± 6.8	−4.4 ± 3.9	−7.3 ± 6.0	0.013^a>b, a>c^
Triglycerides tAUC	240 ± 39	226 ± 27	210 ± 28	0.48
Acetaminophen tAUC	11.7 ± 1.0	13.4 ± 1.2	14.0 ± 1.2	0.02^a<c^

Data are mean values ± SEM. All values in pM × min except glucose and triglycerides in mM × min and acetaminophen in µM × min. *P* values are calculated using repeated measurement ANOVA. *P* values ≤ 0.05 are considered statistically significant. Statistically significant differences between the three treatments are shown using the letters a, b and c, referring to the treatments

*CCK* cholecystokinin, *GIP* glucose-dependent insulinotropic polypeptide (intact), *GLP-1* glucagon-like peptide-1 (total), *iAUC* incremental area under curve, *PYY* peptide YY, *tAUC* total area under curve

**Table 4:** Peak time (pt) and value (pv)

	Olive oil (a)	C4-Dietary oil (b)	Carrot (c)	*P* value
GLP-1 pt	55.38 ± 8.04	57.31 ± 5.68	38.08 ± 11.98	0.27
GLP-1 pv	20.77 ± 1.86	18.77 ± 1.59	13.69 ± 1.33	0.012^a>c^
GIP pt	62.31 ± 5.30	56.92 ± 7.17	43.85 ± 7.08	0.15
GIP pv	68.85 ± 9.24	38.92 ± 7.10	19.54 ± 2.46	< 0.0001^a>b>c^
PYY pt	56.92 ± 11.79	97.31 ± 13.05	40.38 ± 9.24	0.0061^b>c^
PYY pv	93.77 ± 10.77	82.92 ± 12.45	79.08 ± 9.72	0.59
Glucose pt	54.23 ± 8.49	63.08 ± 10.93	49.62 ± 6.06	0.61
Glucose pv	9.89 ± 1.05	10.28 ± 1.13	10.84 ± 1.22	0.0090^a>c^
C-peptide pt	76.92 ± 7.44	85.77 ± 10.54	77.69 ± 10.49	0.51
C-peptide pv	1,918 ± 134.1	1,867 ± 154.0	1,883 ± 152.6	0.86
Insulin pt	60.77 ± 6.09	55.38 ± 5.90	42.31 ± 9.75	0.16
Insulin pv	246.6 ± 20.17	234.2 ± 24.68	232.7 ± 24.14	0.76
Glucagon pt	25.38 ± 4.62	36.92 ± 5.68	22.31 ± 3.03	0.12
Glucagon pv	17.08 ± 2.01	18.31 ± 2.60	16.62 ± 2.07	0.19
CCK pt	55.77 ± 14.38	36.15 ± 6.15	25.77 ± 5.48	0.096
CCK pv	2.71 ± 0.36	2.73 ± 0.40	1.37 ± 0.16	0.0040^a+b>c^
Triglycerides pt	154.6 ± 9.52	110.8 ± 21.32	71.54 ± 17.50	0.0093^a>c^
Triglycerides pv	1.54 ± 0.23	1.37 ± 0.16	1.27 ± 0.18	0.19
Acetaminophen pt	44.23 ± 12.30	43.85 ± 10.27	38.08 ± 5.98	0.69
Acetaminophen pv	0.11 ± 0.01	0.12 ± 0.01	0.13 ± 0.01	0.681

Data are mean values ± SEM. All peak times in minutes, all peak values in pM except triglycerides and glucose in mM and acetaminophen in µM. *P* values are calculated using repeated measurement ANOVA. *P*-values ≤0.05 are considered statistically significant. Statistical significant difference between the three treatments is shown using the letters a, b and c, referring to the treatments

*CCK* cholecystokinin, *GIP* glucose-dependent insulinotropic polypeptide (intact), *GLP-1* glucagon-like peptide-1 (total), *iAUC* incremental area under curve, *PYY* peptide YY, *tAUC* total area under curve

#### Glucose, insulin, C-peptide and glucagon

There were no differences in baseline concentrations or iAUCs for C-peptide or glucose responses, respectively (Fig. 2, Table 3). Insulin iAUC was greater with C4-dietary oil compared to carrot alone (olive oil: 8438 ± 2222 pM × min, C4-dietary oil: 10,690 ± 1671 pM × min, carrot: 4901 ± 1883 pM × min. *P* = 0.02 (Table 3)). Baseline concentrations of glucagon were similar during the three study days. A very slight but higher glucagon response was observed for C4-dietary oil compared to olive oil and grated carrot (olive oil: 17 ± 104 pM × min, C4-dietary oil: 420 ± 95 pM × min, carrot: 18 ± 83 pM × min. *P* = 0.005 (Table 3)).

#### CCK and triglycerides

No differences in baseline concentrations were observed between the three experimental days for CCK or triglycerides, respectively (Table 2). Olive oil elicited a greater rise in triglyceride concentrations whereas C4-dietary oil and carrots were without effect (olive oil: 12.0 ± 6.8 mM × min, C4-dietary oil: −4.4 ± 3.9 mM × min, carrot: −7.3 ± 6.0 mM × min. *P* = 0.013 (Table 3, Fig. 2)). A higher CCK response was seen following olive oil compared to grated carrot (olive oil: 103 ± 34 pM × min, C4-dietary oil: 33 ± 37 pM × min, carrot: −4 ± 22 pM × min. *P* < 0.01 (Table 3, Fig. 2)). There was no difference for iAUC_CCK_ between olive oil and C4-dietary oil, and both elicited peak values that were higher than the control meal with only carrot (olive oil: 2.71 ± 0.36 pM, C4-dietary oil: 2.73 ± 0.40 pM, carrot: 1.37 ± 0.16 pM. *P* = 0.0040 (Fig. 2, Table 3, Table 4)).

#### Gastric emptying rate

Gastric emptying rate estimated as the tAUC for acetaminophen, was lower with olive oil compared to the carrot meal (olive oil: 11.7 ± 1.0 µM × min, C4-dietary oil: 13.4 ± 1.2 µM × min, carrot: 14.0 ± 1.2 µM × min. *P* = 0.02 (Table 3)).

## Discussion

Our in vitro data demonstrate that C4-dietary oil by itself cannot activate GPR119, in accordance with the observation that a hydroxyl group in the lipid molecule is required for agonist activity on GPR119.^15^ This indicates that C4-dietary oil, like other triglycerides, must be hydrolyzed before it can activate nutrient sensors.^41, 42^ Several different lipids (lysophopholipids, acylamides, 2-monoacylglycerols) can act as agonists for GPR119 in vitro.^15, 43^ We have shown that 2-OG stimulates GPR119 not only in vitro, but probably also in vivo where direct administration of 2 g 2-OG in the intestine of healthy subjects resulted in an increase in plasma GLP-1 within 25 min after administration.^16^ Oleic acid in an amount corresponding to that present in 2 g 2-OG did not stimulate GLP-1 release, suggesting that the meal-related formation of 2-monoacylglycerols acting on GPR119 during fat ingestion is indeed responsible for a significant part of the GLP-1 response. Recently, we have shown that compared to 20 ml (19 g) olive oil, an equimolar amount of 1,3-dioctanoyl 2-oleoyl glycerol (13.15 g, 21.6 mmol) was just as effective with respect to GLP-1 release in humans, suggesting that the stimulating factor in the consumed olive oil may be mainly 2-OG acting on GPR119.^12^ Studies of a mouse model involving self-administration of fat emulsions also demonstrated the mice can sense 2-OG in the gastrointestinal system probably via GPR119 activation.^44^

The present in vivo study was designed to characterize the effect of C4-dietary oil on incretin hormone secretion compared with equimolar amounts of olive oil and with grated carrots, respectively, in overweight patients with T2D. By examining the same individuals in a cross-over design inter-individual variation was minimized. Patients were naturally more well treated due to exclusion criteria, but they were recruited from diabetes outpatient clinics in order to reflect reality well. Although fatty acids generated from the digestion of olive oil may stimulate incretin release via GPR40 and GPR120^7, 8^ or other mechanisms,^9^ they are also rapidly absorbed together with 2-monoacylglycerol and subsequently re-esterified. Apparently, at a dose of 20 ml olive oil to healthy human subjects, the long-chain fatty acid moiety of olive oil seems to play a minor role in GLP-1 release, while it seems to have an additive effect together with 2-OG on GIP release and be of major responsibility for CCK release.^12^ In the present study, C4-dietary oil also gave the same GLP-1 response as olive oil, further supporting the GLP-1-releasing action of 2-OG, while the C4 fatty acid (butyrate) seems to have no effect, as judged from the finding that an oral intake of an equimolar amount of tributyrin did not stimulate GLP-1 release.^12^ The iAUCs for GIP on the olive oil-day and the C4-dietary oil day were not different although the peak value for GIP was clearly higher on the olive oil-day. In our previous study, we found that the GIP response to olive oil can be explained by a combined stimulatory effect of both 2-OG and oleic acid,^12^ and the present data tend to support this conclusion. Since the incretin responses to 19 g of olive oil (171 kcal) and only 10.7 g of C4-dietary oil (81 kcal) were similar, this indicates that C4-dietary oil, per unit of energy content, is more effective than olive oil in releasing GLP-1 and probably also GIP.

Neither olive oil nor C4-dietary oil had effects on iAUC for PYY, although our previous study demonstrated a clear PYY-stimulating effect of equimolar doses of both C8-dietary oil and olive oil in healthy subjects.^12^ Whether this difference in PYY responses is due to testing the oils on overweight patients with T2D as compared to the previous study, where they were tested on healthy subjects is at present not clear. However, Fernandez-Garcia et al.^45^ has shown that in morbidly obese subjects, both insulin resistance and abnormal glucose metabolism impair GLP-1 and PYY responses to a 60 g fat load. Also, the 2-OG receptor GPR119 has been reported to be involved in stimulating PPY release.^46^

In the present study, both oils stimulated CCK release with olive oil being more efficacious than C4-dietary oil, while in our previous study C8-dietary oil and tributyrin did not stimulate a CCK response.^12^ One explanation for these differences in CCK responses between the C8-dietary oil study^12^ and the present C4-dietary oil study may possibly be related to the rate of gastric metabolism of these two structured triglycerides. Due to the short-chain fatty acids in C4-dietary oil it may have been metabolized more rapidly in the stomach by gastric lipase than C8-dietary oil. This would result in the generation of 2-OG having longer time to undergo spontaneous acyl migration^47^ before entering the intestine, thereby resulting in formation of 1-oleoyl glycerol, which is substrate for hydrolysis by both gastric lipase and pancreatic lipase.^48^ This would result in a larger proportion of free oleic acid generated from C4-dietary oil than from C8-dietary oil. Long-chain fatty acids are known to stimulate CCK release.^49, 50^

GLP-1 secretion may be stimulated by CCK.^51^ However, in our study we saw a GLP-1 release in the C4-dietary oil group after 75 min when CCK levels were low. In earlier studies, we did not observe increased GLP-1 secretion after CCK administration, however,^52^ so in support of our earlier studies^12, 16^ it seems that a GLP-1 release induced by 2-OG can be elicited without involving CCK release.

Administration of all three meals was followed by an increase in the PG (no difference among meals) reflecting digestion of the carbohydrates in carrots.^12^ There was a tendency that the oil meals elicited higher C-peptide responses (Fig. 2). However, the greater increase in both GLP-1 and GIP following ingestion of oils compared to carrots was not sufficient to influence insulin secretion in these experiments. GLP-1 is known to increase insulin secretion in a dose-dependent manner.^53–55^ In a previous study, GLP-1 infusions resulting in peripheral plasma GLP-1 concentrations of ∼50 pM stimulated insulin secretion at fasting glucose levels, but in the current study, the increase of GLP-1 from 8 to 15 pM might be too small to influence insulin secretion significantly.^53^ We know from previous studies^55^ that in order to demonstrate the insulinotropic effect of GLP-1 and GIP at fasting PG concentrations, it is necessary to clamp PG most thoroughly. That was unfortunately not done in the current study. We also know that portal insulin levels can be up to six times higher than the peripheral levels given that the flow of the splanchnic circulation is one third of the systemic and that the hepatic insulin extraction is 50%. Therefore, we cannot say that the incretin release was insufficient in this study. On the contrary, these data suggest an effect due to the relatively low glucose values on oil days.

In animal studies in which GPR119 agonists were administered in high doses without influencing glucose concentrations,^56^ stimulation of the GPR119 also had no insulinotropic activity at lower glucose concentrations. In addition, the lack of acute effect on insulin secretion in our study may be due to the decreased sensitivity of the beta cells to incretin hormones in T2D, as shown by Højberg et al.^57^ In that study, no increase in insulin release following intravenous infusion of GLP-1 and GIP (resulting in physiological plasma concentrations), respectively, was observed.

Gastric emptying might have influenced the results observed in this study. Our results show that olive oil inhibited gastric emptying. In our previous study,^12^ we saw no clear effect of the same dose of 20 ml olive oil on gastric emptying in healthy subjects. Gastric emptying rate was evaluated using the acetaminophen absorption test. The test is an indirect assessment of gastric emptying, and it is well validated against scintigraphic methods considered the gold standard for measurements of gastric emptying.^58, 59^ In the present study, there were no differences in gastric emptying between C4-dietary oil and carrot alone, suggesting that fatty acids from olive oil may contribute to the effect on gastric emptying in these overweight patients with T2D.

Recently, it has been shown that diabetic mice lacking the short-chain receptors, GPR41 and GPR43, have increased insulin secretion and improved glucose tolerance^60^ thereby questioning the beneficial metabolic role of dietary short chain fatty acids via this pathway, although prolonged oral intake of butyrate in diet-induced obese mice resulted in increased insulin sensitivity and increased energy expenditure.^23^ Anyhow, both C4-dietary oil and C8-dietary oil increased GLP-1 release just as efficaciously as equimolar amounts of olive oil, which provided more calories. Further studies evaluating chronic administration of C4-dietary oil or C8-dietary oil for a longer period should therefore be carried out, since beta cell sensitivity to incretin hormones may improve during anti-diabetic therapy.^49^

In conclusion, our study suggests that postprandial GLP-1 and GIP responses in overweight patients with T2D may be augmented by GPR119 activation. C4-dietary oil had on a molar basis the same activity as olive oil. However, if one calculates by unit of energy, C4-dietary oil may be more potent as incretin releaser than olive oil and could possibly be useful for some dietary products.
